# Supplementary material for: A Comprehensive Evaluation of Generalizability of Deep Learning-Based Hi-C Resolution Improvement Methods
Source: Genes (Basel). 2023 Dec 29;15(1):54. doi: 10.3390/genes15010054 (PMC10815746; doi:10.3390/genes15010054)
Supplement: Supplementary file 1 [file genes-15-00054-s001.zip › genes-2780807-supplementary_materials.pdf]

Investigating the performance of deep learning methods for Hi-C resolution improvement

Supplementary Material

S1. Retraining of HiCNN, HiCNN2 and HiCPlus

We retained the original implementation of these methods wherever we could. We changed the output objective from predicting raw counts to predicting a matrix with values between 0-1, similar to DeepHiC, and for these three, we replaced the SGD optimizer with the Adam optimizer to stabilize the training process and improve the performance. In the figures **??** we show the training curve for these methods for all the four version we retrained.

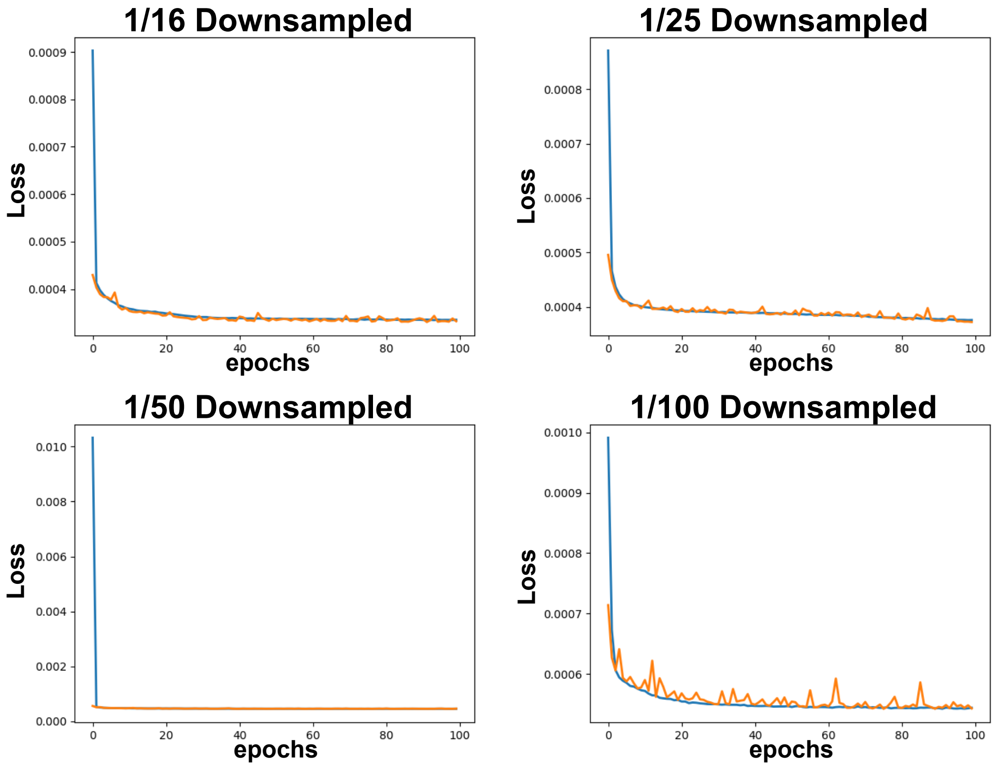

Figure S1. This figure shows the loss curves for HiCPlus models on four version we trained with each downsampling ratio. We use the version of model (across all the epochs) that minimizes the validation loss.

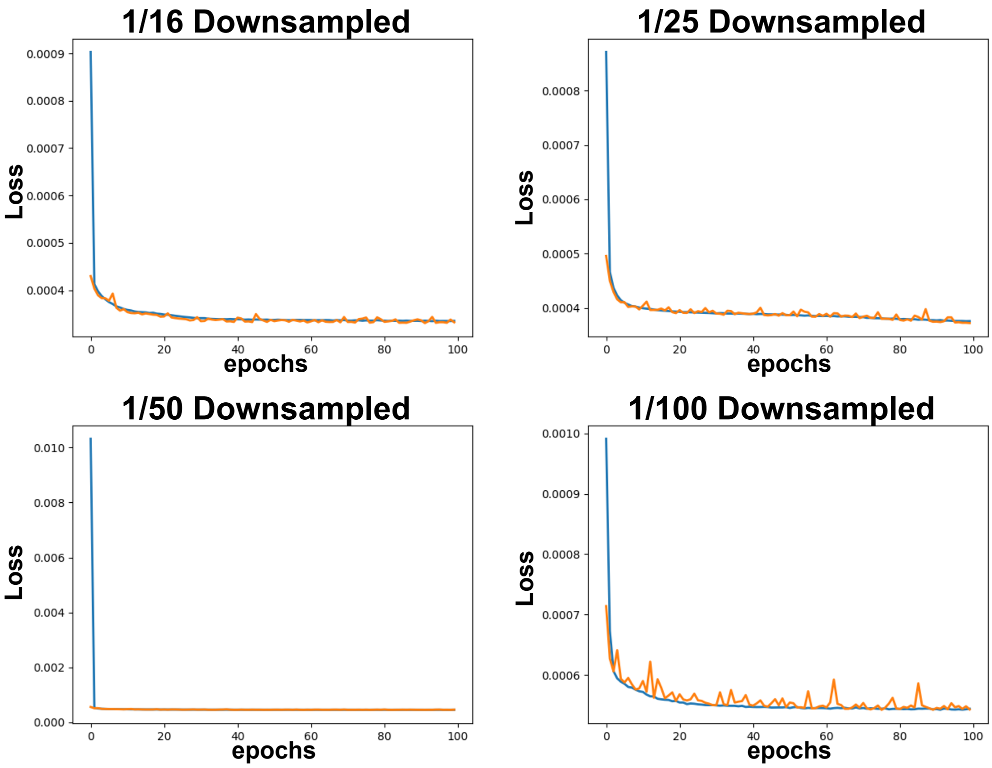

Figure S2. This figure shows the loss curves for HiCNN models on four version we trained with each downsampling ratio. We use the version of model (across all the epochs) that minimizes the validation loss.

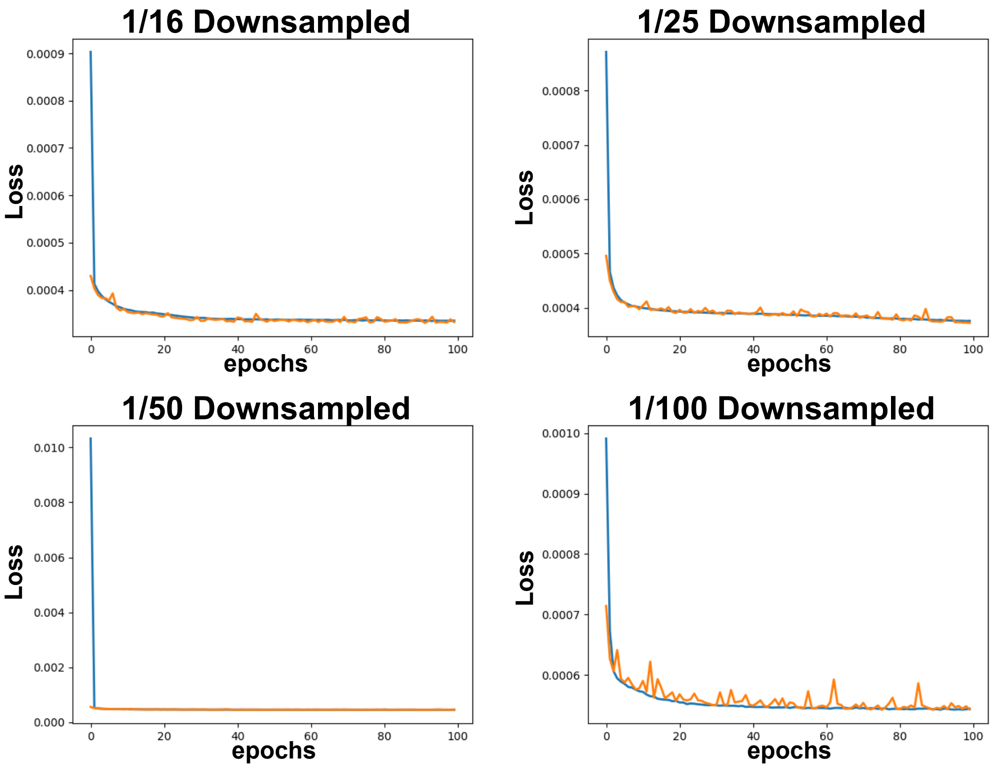

Figure S3. This figure shows the loss curves for HiCNN2 models on four version we trained with each downsampling ratio. We use the version of model (across all the epochs) that minimizes the validation loss.

S2. Details of the evaluated methods

- **Gaussian Smoothing** We applied the Gaussian Smoothing filter, commonly used as a baseline [6], to establish emphasize the performance benefits of the deep-learning-based methods. This method uses a 2D kernel of shape $n \times n$ where n is a hyperparameter. Each kernel value follows a 2D Gaussian distribution with hyperparameters σ_x and σ_y that represent the relative importance of neighboring features in prediction. The smoothing operation convolves this kernel on each pixel of a 2D image, or in our case, a HiC matrix read count, to update its value. This updated value contains the average of the neighboring values weighted by 2D Gaussian distribution in the kernel. This smoothing operation removes noise in the input matrix and improves the peak signal to noise ratio (PSNR) at the cost of blurring the features. For our experiments, we performed a grid search and found the kernel size of $n = 17$ and $\sigma_x = \sigma_y = 7$ to give the best reproducibility score on the validation set of LRC HiC matrices.
- **HiCPlus [6]** HiCPlus is the first application of deep learning to improve HiC resolution. It utilizes a standard three-layer convolutional neural network (CNN) architecture to upscale a low-resolution HiC matrix by mapping it to the target high-resolution matrix. To optimize its parameters, HiCPlus uses a mean squared error (MSE) loss. HiCPlus inputs HiC matrices as sub-matrices of size 40×40 binned at 10Kbp resolution and taken from 2 Mbp distance from the diagonal. All the following deep learning-based models follow the same input formulation. For this study, we make certain modifications to the original code to make HiCPlus comparable to the more recent implementations. For example, the original HiCPlus was trained to generate raw read counts rather than normalized HiC matrices. Therefore, we retrain the model to work with normalized high-resolution and low-resolution pairs of HiC sub-matrices. Moreover, the original implementation was trained to upscale only the matrices that had been downsampled to $\frac{1}{16}$ of the read counts of a high-resolution HiC map. We retrain the HiCPlus with three additional input HiC datasets with $\frac{1}{25}$, $\frac{1}{50}$, and $\frac{1}{100}$ downsampling ratios to explore the performance on matrices beyond the original downsampled version.
- **HiCNN [8]** HiCNN model also uses a CNN architecture like HiCPlus, except that it consists of a much deeper 54-layer neural network with ResNet layers [18]. The choice of ResNet layers provides two key benefits - (1) it provides additional architectural complexity to learn relevant non-linear relationships between the inputs and the outputs, and (2) they train significantly faster than regular CNN layers saving substantial time during training. In addition, the skip connections in ResNet layers further avoid model overfitting on the data. HiCNN, similar to HiCPlus, uses an MSE loss to optimize its parameters and learn the mapping between the input low-resolution matrix and the high-resolution target matrix. It also produces raw read counts and is trained with downsampling ratios of $\frac{1}{8}$ and $\frac{1}{16}$ and $\frac{1}{25}$. Therefore, for consistency, we retrain the model with additional datasets with downsampling rations of $\frac{1}{50}$ and $\frac{1}{100}$ and standardized values.
- **HiCNN2 [9]** HiCNN2 extends the architecture of HiCNN by ensembling multiple methods to achieve better resolution. There are three different ConvNets in HiCNN2; the first ConvNet is similar to HiCNN in terms of how it uses global and local residual learning; it also concatenates features across residual blocks to improve performance. The second ConvNet is a modified version of VDSR which only uses the global residual learning. The third ConvNet is the HiCPlus model. Each model produces a 28x28 output matrix that is then combined through weights tuned during the training process. This method, similar to HiCPlus and HiCNN, also predicts raw read counts and is only trained with $\frac{1}{8}$ and $\frac{1}{16}$ and $\frac{1}{25}$ downsampling ratios. Similar to both HiCNN and HiCPlus, we retrain HiCNN2 to predict normalized read counts across all four downsampling ratios.

- **HiCGAN [11]** HiCGAN paper argues that the Mean Squared Loss function used in both HiCNN and HiCPlus causes these models to generate over-smooth matrices. Therefore, it proposes using a Generative Adversarial Network (GAN) model for the HiC resolution improvement task. A GAN architecture consists of (1) a generator and (2) a discriminator. The generator's objective is to produce data that increasingly resembles the original distribution, and the goal of the discriminator is to identify fake (generated) data from the original data. This coupled training causes both models to get iteratively better at their tasks. HiCGAN uses a specialized form of GAN, which is called the conditional GAN (cGAN). In cGANs the generator produces an output conditional on the provided input instead of a random noise input. To optimize the parameters in the model, HiCGAN uses the discriminator loss and the MSE loss to generate matrices that are highly similar to the target HiC matrices. The HiCGAN paper shows that this method produces better-quality HiC matrices with sharper and more prominent features than the previous method.
- **DeepHiC [10]** DeepHiC paper, like HiCGAN [11], argues that the Mean Squared Loss function used in both HiCNN and HiCPlus causes these models to generate over-smooth matrices. Therefore, it uses Generative Adversarial Network (GAN) model for the HiC resolution improvement task. A GAN architecture consists of (1) a generator and (2) a discriminator. The generator's objective is to produce data that increasingly resembles the original distribution, and the goal of the discriminator is to identify fake (generated) data from the original data. This coupled training causes both models to get iteratively better at their tasks. DeepHiC substantially revises the previously proposed loss functions to contain additional functions that include Total Variation Loss and Perceptual Loss. These loss function along side Mean Squared Error Loss and Discriminator Loss causes the model to generate matrices with sharper features that are more biologically informative [10]. The paper also shows that training the deep learning models on standardized HiC matrices improves their performance further. Moreover, the paper trains the DeepHiC model with a downsampling ratio of up to $\frac{1}{100}$ to have model weights available for even the sparsest real-world HiC matrices.
- **VeHiCLE [13]** VeHiCLE is another GAN-based model like HiCGAN and DeepHiC. However, it makes additions to both the model architecture and loss functions used while training. Apart from using a GAN architecture, it also trains a variational auto-encoder (VAE). The output obtained by passing the HiC matrices through the trained VAE is used in a loss function to train the GAN. This loss obtained from the VAE is called the variation loss. VeHiCLE also uses the adversarial and MSE losses seen in previous methods. However, it introduces yet another loss called insulation loss. The insulation loss is a biologically inspired loss that utilizes insulation scores used to identify TADs in a HiC contact matrix. VeHiCLE is trained on input and target matrices of sizes 269×269, unlike the previous methods that used 40×40 sub-matrices as inputs. The paper shows that this increased matrix size improves performance, thus hypothesizing that the 40×40 matrices are too small to adequately capture information about large-scale HiC features (such as TADs). We created new datasets for VeHiCle that had HiC sub-matrices of size 269x269 to ensure a fair comparison of VeHiCle with other deep learning based methods.

S3. Details of the evaluation metrics

- **Structural Similarity Index Measure (SSIM)** Structural Similarity Index Measure (SSIM) is a metric that measures the perceived perceptual quality of an image against an original undistorted and higher quality image. SSIM measures this perceptual quality by comparing the luminance, contrast, and structural properties in small local regions of the images. A weighted sum of these properties allows SSIM to assign a similarity score that closely mimics the way humans perceive differences in images. However, we postulate that the HiC contact maps should be compared based on

their underlying biological properties instead of their visual similarities. Therefore, assigning a similarity score based on SSIM score may hold little biological relevance and might lead to misleading conclusions about the quality of the generated datasets.

- **Pearson Correlation Coefficient (PCC)** Pearsons Correlation Coefficient (PCC) is a linear measure of the correlation between two sets of data distribution. PCC is a ratio of covariance between two variables and its product with their standard distribution. This metric essentially measures covariance between the two datasets, normalized to have a value between -1 and 1. Here, -1 or 1 values imply highly negatively or positively correlated, respectively, and a 0 value implies no correlation between the two datasets.
- **Spearman's rank Correlation Coefficient (SCC)** Spearman's rank Correlation Coefficient (SCC) measures the statistical dependence between the rank of two variables. This measure essentially captures how well two variables can be described using a monotonic function. SCC between two variables is equal to the PCC of the rank of variables. Thus, SCC has a value of +1 or -1 when either of the variables is a perfect monotone of the other. It has a value of 0 when they do not correlate monotonically.
- **Hi-C-Rep [?]** Hi-C-Rep measures the reproducibility between two Hi-C datasets based on spatial features such as distance dependence and domain structure. To enhance the domain structure in the Hi-C matrices, Hi-C-Rep applies a mean filter, which filters out the stochastic noise that can potentially arise from the experimental protocols and possibly also through low read counts in the dataset. Hi-C-Rep then stratifies the read counts in the matrix based on their distance and measures the correlation between each stratum. The correlations between each stratum are then combined using a weighted average of each stratum, where the weighting coefficients are calculated using the Cochran-Mantel-Haenszel (CMH) statistic. Hi-C-Rep reports a score between -1 and 1, similar to Pearsons's Correlation and Spearman's, with scores close to 1 representing high similarities between the Hi-C matrices.
- **GenomeDISCO [20]** The GenomeDISCO method focuses its correlation analysis on the property that high-level order structures (loops and compartments) at multiple scales between two Hi-C contact maps are similar if they are highly correlated. To leverage that, GenomeDISCO measures the correlation over a range of genomic scales by smoothing the Hi-C matrices at different intensities. GenomeDISCO uses Random Graph walks to smooth out the matrices by formulating the Hi-C data as a Graph. In this contact matrix graph, each node represents a genomic region and, edge weights represent the contact value between these regions. Random Graph walk measures the probability of finding a path between any given node pair i and j given we can only take t steps, where for each step t, the edge chosen for the walk is dependent on edge weight. GenomeDISCO then raises the power of each value in the random walk network by power of t to construct a smoothed contact map. The higher the value of t, the higher dimensional genomic features(such as A/B compartments) the smoothed contact map summarizes. Finally, to obtain the reproducibility score, GenomeDISCO computes the area under the curve of the t against the L1 distances between the smoothed contact maps. Since maximum L1 distance between contact maps can be 2, the reproducibility is calculated by $score = 1 - combined - distance$ which gives the score in range $[-1, 1]$. Where value of 1 represents a high similarity between the input contact maps.
- **Hi-C-Spector[?]** Hi-C-Spector computes the correlation by performing a spectral analysis of the input Hi-C matrices. Hi-C-Spector's analysis is centered around the observation that the first two eigenvectors of the Hi-C matrix correspond to the higher dimensional structures such as A/B compartments. Given that observation, two Hi-C matrices that are similar should have eigenvectors that are also similar. To compute the similarity between those eigenvectors, Hi-C-Spector does the following operations: First of all, it constructs a Laplacian Matrix L by subtracting the Diagonal of the contact matrix D from the input contact matrix W. In the second step, it normalizes

the contact map by applying a transformation $D^{-1/2}LD^{-1/2}$. In the third step, Hi-C-Spector decomposes the normalized matrix into its eigenvectors. It keeps the first two eigenvectors and discards the rest because those vectors represent the noise in the data. In the last step, Hi-C-Spector computes a summation of pair-wise distances of the Eigenvectors of the input Hi-C contact map against the reference Hi-C map and normalizes it in the range 0-1 to assign a similarity score, where the score of 1 represents the high similarity between two contact maps.

- **QuASAR-Rep [?]** QuASAR-Rep bases its correlation analysis on the observation that in a distance matrix, as the distance between two features approaches zero, the correlation between rows of these features approaches one. QuASAR-Rep utilizes this property to compute the correlation between two Hi-C samples. It first filters out all the intra-chromosomal contacts from the input Hi-C samples and then all the rows that do not contain a non-zero contact bin within a 100 bin range of the diagonal. Next, it computes the background signal-to-distance ratio by taking the average reads of each inter-bin distance. Next, it sets up the interaction correlation matrix. The interaction correlation matrix is constructed based on all the pairwise sets of rows and columns (within a range of 100 bins of each other) in the log-transformed enrichment matrix. The values in the enrichment matrix are non-filtered counts divided by background signal to distance values. Finally, to compute the correlation between a given set of rows A and B, correlation is calculated between all the columns in a 100 bin range of both rows (excluding the filtered rows). QuASAR-Rep adds 1 to all the valid entries and takes a square root to construct the interaction matrix. The weighted interaction matrix is just an element-wise product of the correlation matrix and the interaction matrix divided by the sum of all valid interaction matrix entries. It calculates the final reproducibility score by computing the correlation between the weighted correlation matrices of the input samples. This score is 0 to 1, where one represents that Hi-C inputs are highly similar and zero represents that they are highly dissimilar.

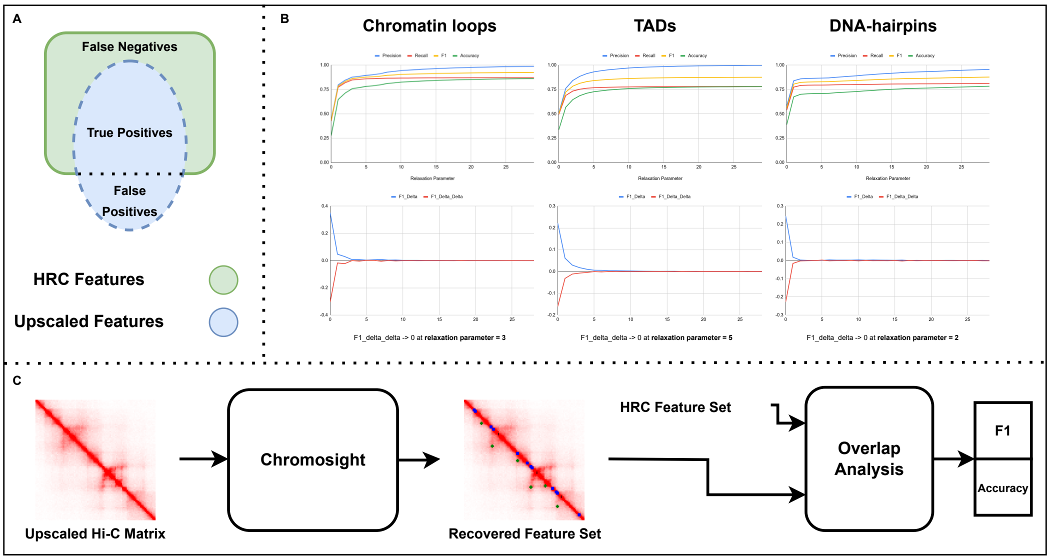

Figure S4. Overview of our Biological Feature Analysis Pipeline: A We define True Positives as features that we find to be overlapping between the upscaled Hi-C matrix and the HRC matrix. Similarly, we define features that are present in the HRC matrix and not in the upscaled Hi-C matrix as False Negatives. Conversely, features we find in upscaled matrix and not in the HRC matrix as the false postives. We compute F1 scores using these definitions. **B** We optimize the parameter "r" (relaxation paramter) for each feature, chromatin loops, TADs and DNA-hairpins. This relaxation parameter defines the overlapping radius between the position of features between the upsampled and HRC features. We tune this paramter by comparing features between the GM12878 HRC and GM12878 biological replicate cell lines and find the value of r that maximizes the F1 score while keeping the value of r small. We found a value of 3 for chromatin loops, 5 for TADs and 2 for DNA-hairpins. **C** We summarize our biological feature analysis pipeline, we first extract biological features using Chromosight and then compute F1 scores.

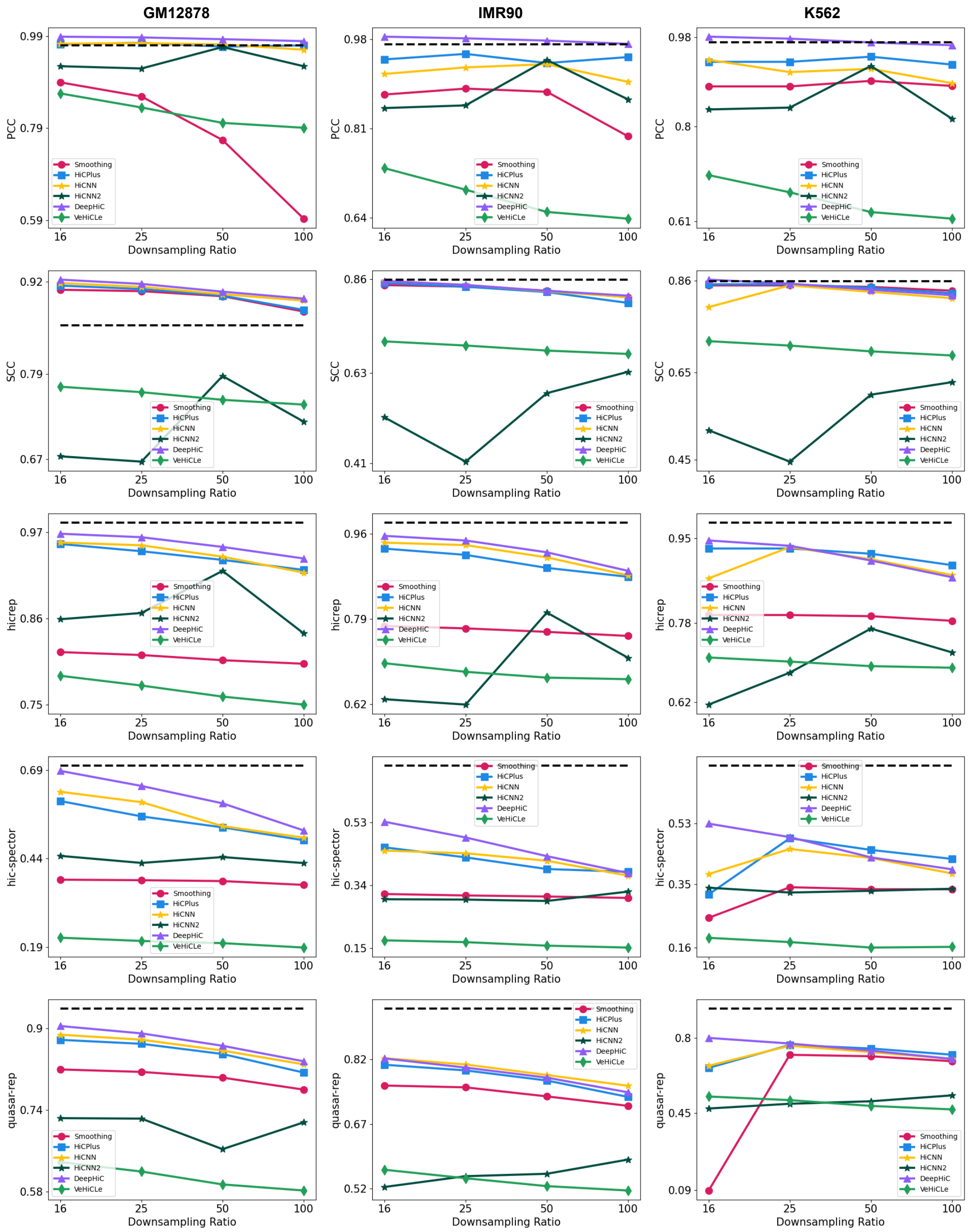

Figure S5. This figure shows the performance of deep-learning based Hi-C upscaling methods on computationally downsampled methods on four downsampling ratios (shown on x-axis), three cell-lines five metrics. All methods except VeHiCLe show performance very similar to the replicate shown as black dotted bold line.

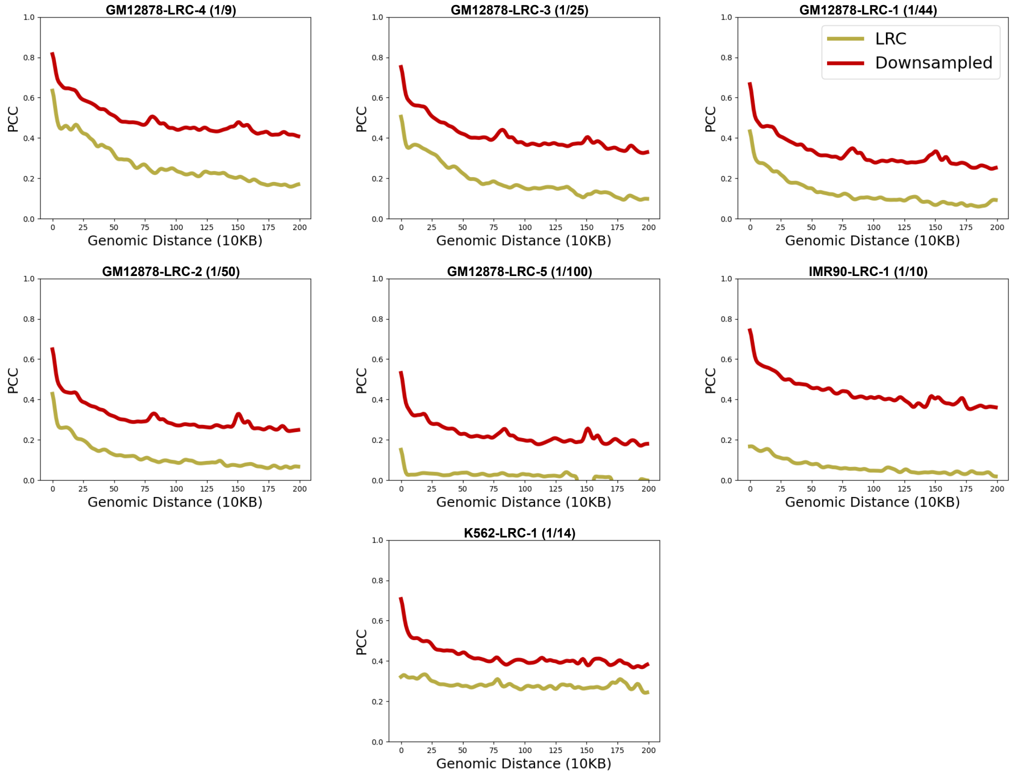

Figure S6. Our results on comparing the PCC (y-axis) across various genomic distances (x-axis) suggest that LRC datasets show a smaller similarity with HRC Hi-C dataset in both increasing levels of read sparsity and in cross-cell-type cases.

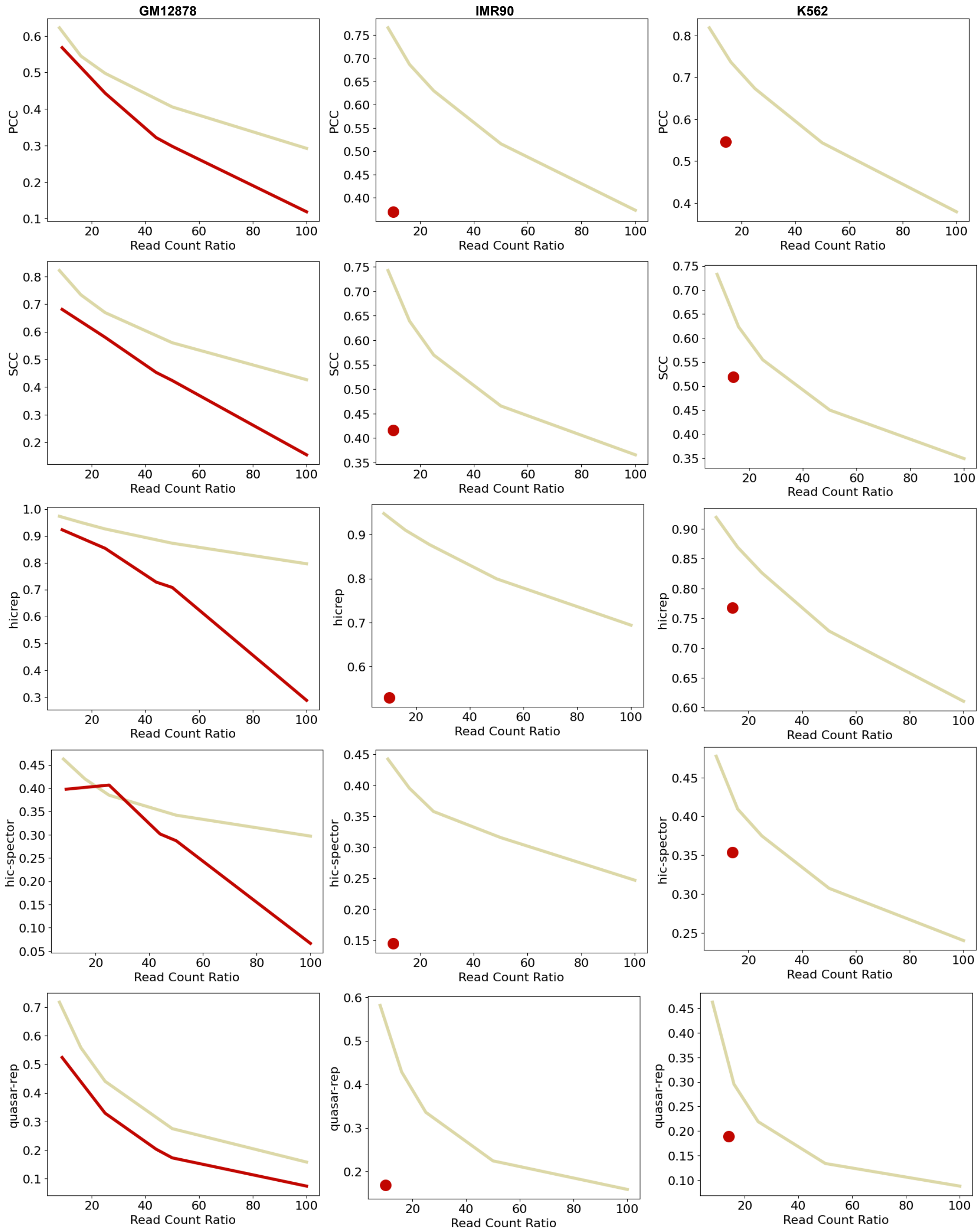

Figure S7. Our results on comparing the PCC (y-axis) across various genomic distances (x-axis) suggest that LRC datasets show a smaller similarity with HRC Hi-C dataset in both increasing levels of read sparsity and in cross-cell-type cases

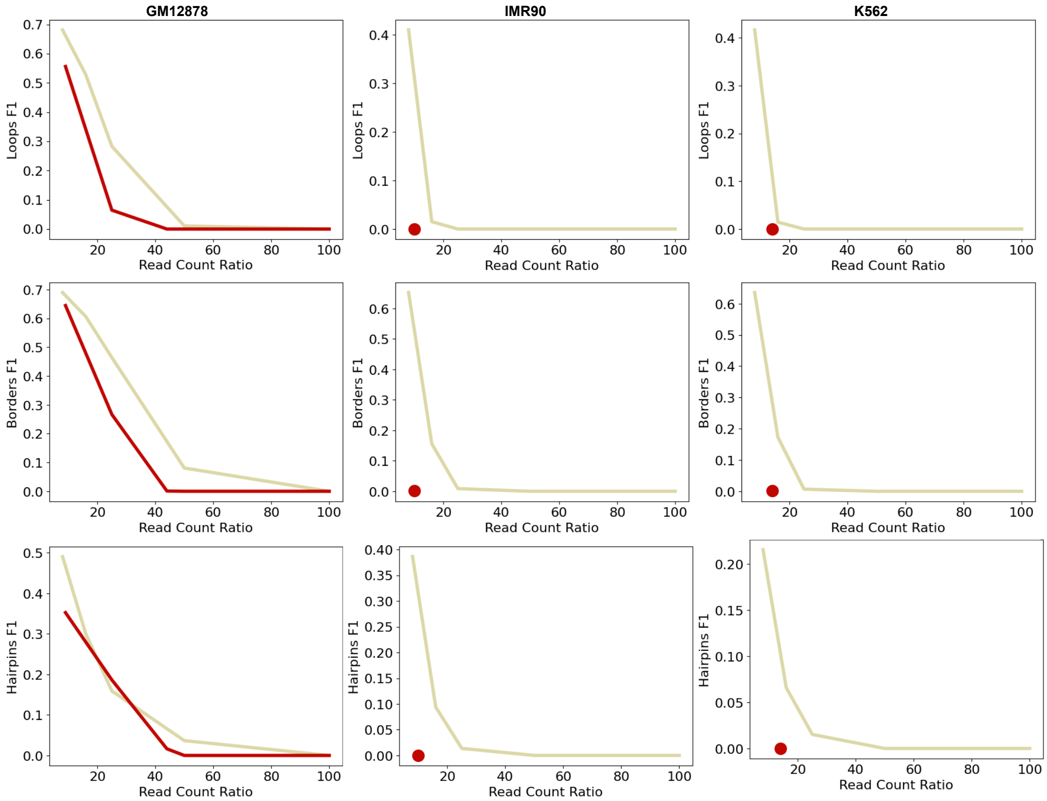

Figure S8. We show the impact of the distributional difference in the structural information contained in downsampled and experimentally generated LRC Hi-C contact maps by comparing scores across both correlation and Hi-C similarity metrics

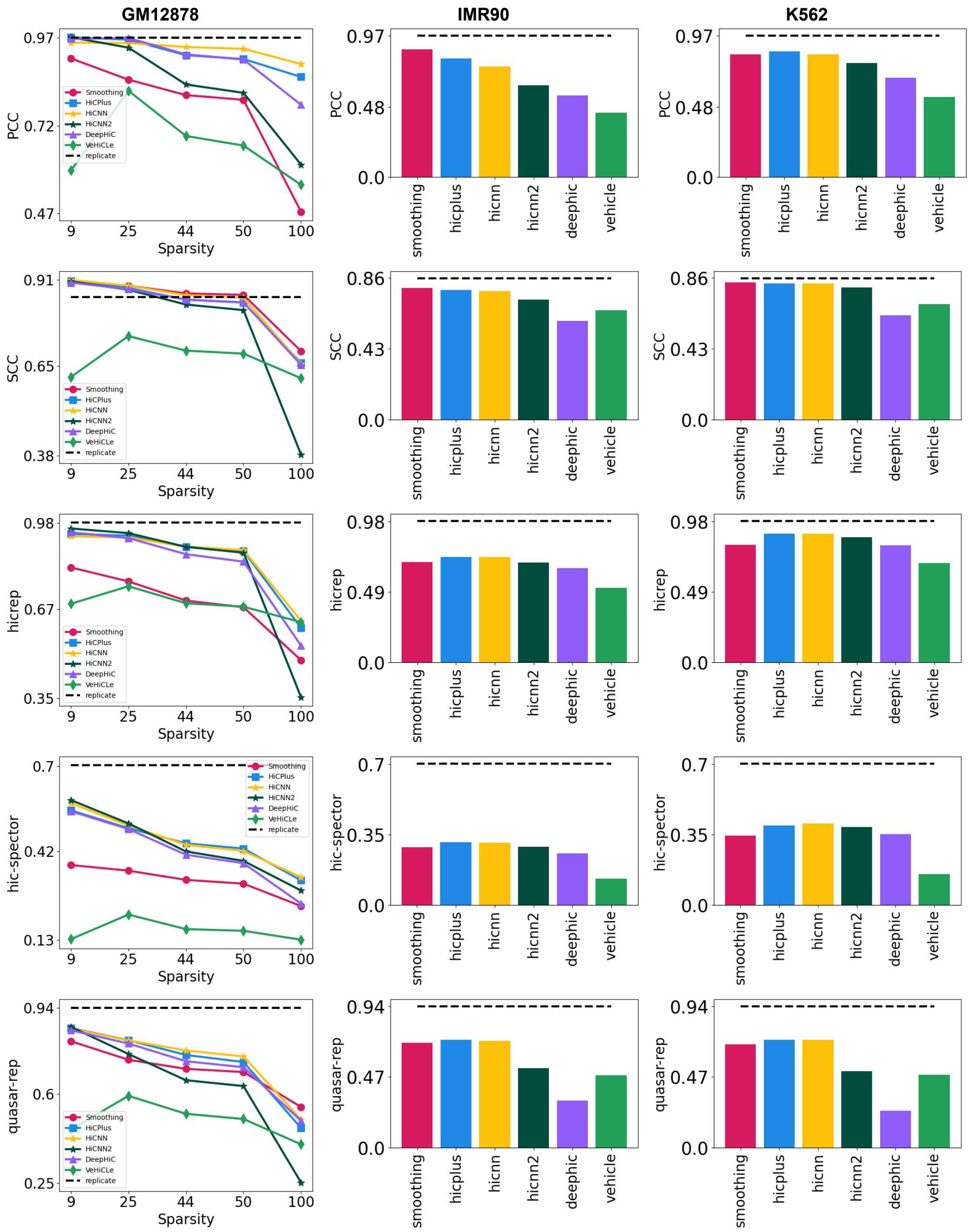

Figure S9. This figure shows performance on rest of the metrics, and our results highlight that there is a reduction in performance as the sparsity increases in GM12878 datasets and also the results in a cross-cell-type (IMR90 and K562) setting.

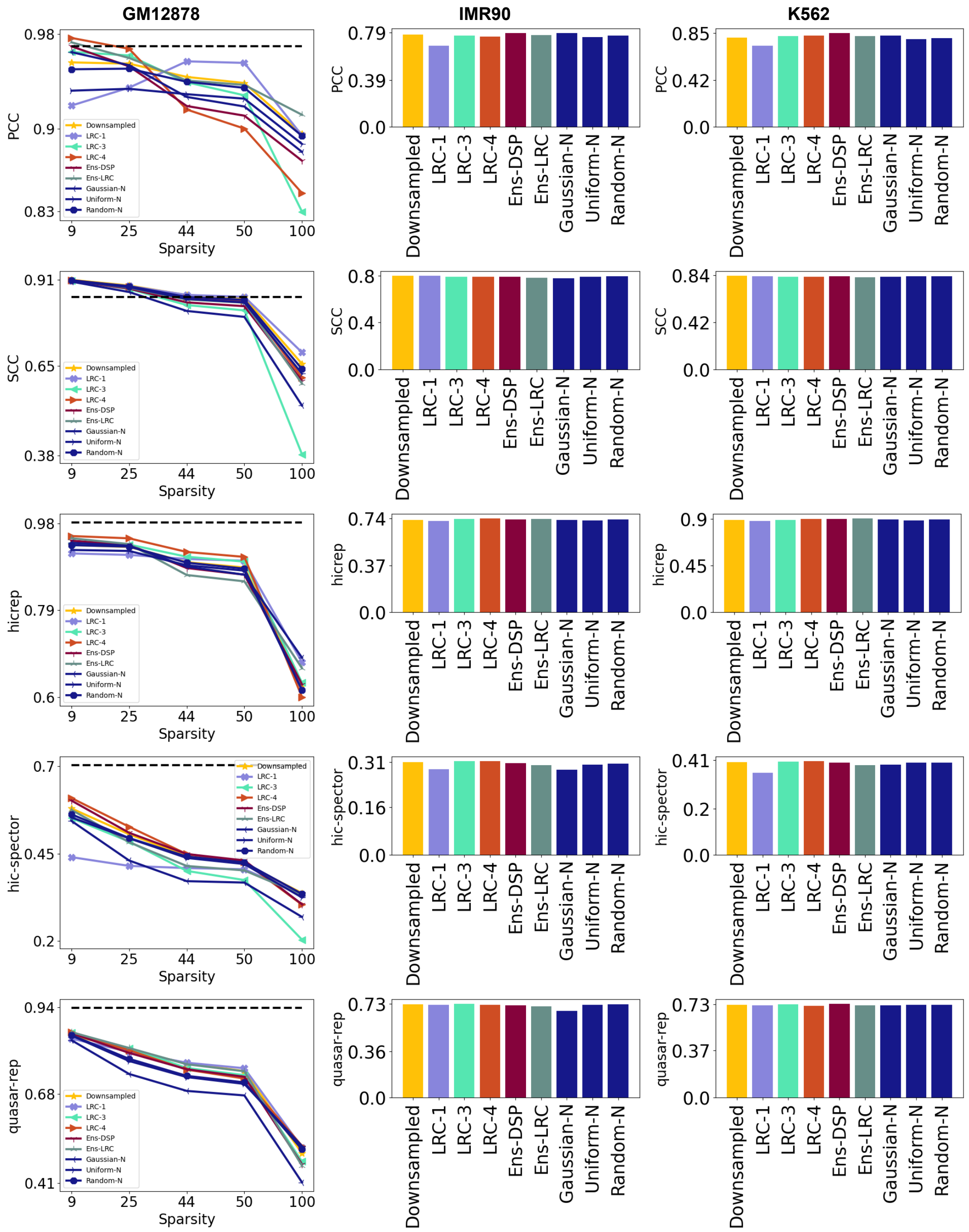

Figure S10. We show results of retraining across five additional metrics and also across additional retraining variants, such as downsampled datasets augmented with noise
